# Correction to: Preclinical assessment of transiently TCR redirected T cells for solid tumour immunotherapy

**DOI:** 10.1007/s00262-019-02409-6

**Published:** 2019-11-27

**Authors:** Nadia Mensali, Marit Renée Myhre, Pierre Dillard, Sylvie Pollmann, Gustav Gaudernack, Gunnar Kvalheim, Sébastien Wälchli, Else Marit Inderberg

**Affiliations:** 1 Department of Cellular Therapy, Department of Oncology, Oslo University Hospital, The Norwegian Radium Hospital, 0379 Oslo, Norway; 2 Department of Cancer Immunology, Institute for Cancer Research, Oslo University Hospital, The Norwegian Radium Hospital, 0379 Oslo, Norway; 3 Faculty of Medicine, University of Oslo, 0316 Oslo, Norway

## Correction to: Cancer Immunology, Immunotherapy (2019) 68:1235–1243 10.1007/s00262-019-02356-2

The original version of this article unfortunately included a mistake in Fig. 2b where the images of mice in the tumour control group (right), day 30 (bottom) should be removed as the wrong images (duplicate of day 17) were inserted by mistake. At this time point the tumour control mice were no longer alive and the images were replaced by black areas.

The revised Fig. 2 is placed on the following page.

**Fig. 2:**
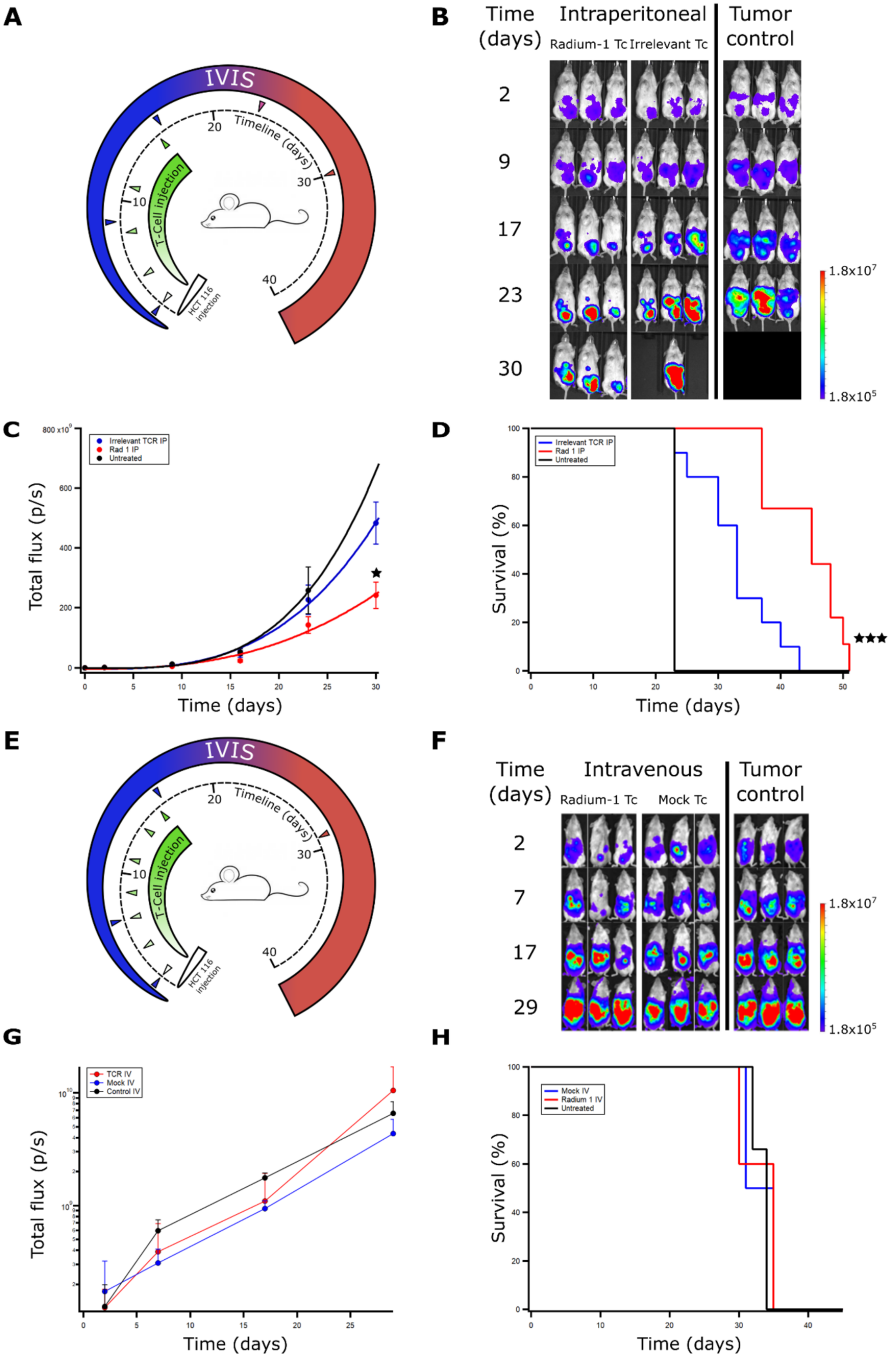
In vivo efficacy of transiently transfected T cells depends on route of administration. (**a**) NSG mice were injected i.p. with 10^6^ HCT 116 ff-Luc 2 days before injection of T cells. T cell treated groups were treated on days 2, 4, 8, 11, and 16 with Radium-1 TCR or irrelevant TCR expressing T cells i.p. (*n* = 10). The Radium-1 TCR group was treated with 10^7^ Radium-1 TCR electroporated T cells at each injection, the irrelevant TCR group (*n* = 10) was treated with 10^7^ DMF5 TCR electroporated T cells at each injection i.p. Tumour control received no treatment (*n* = 4). Three mice from each group are shown. (**b**) Tumour load was evaluated by bioluminescence imaging on days 2, 9, 17, 23, and 30. Black areas indicate loss of mice. (**c**) Average tumour load (total flux photons/s) in the different groups are shown (*p* = 0.031 and 0.0089). (**d**) Survival time of the different treatment groups after tumour injection (*p* = 0.0072). The results shown are representative of three independent experiments. (**e**) Radium-1 Tc treated group (*n* = 5) and mock electroporated Tc treated group (*n* = 4) were treated with 10^7^ T cells i.v. at indicated time points. The tumour control group (*n* = 3) was not given any treatment. (**f**) Tumour load was evaluated by bioluminescence imaging on days 2, 7, 17, and 29. (**g**) Average tumour load (total flux photons/s) in animals given T cells i.v. and control group, (**h**) Kaplan–Meier animals given T cells i.v. and control group

